# Multimodal Prehabilitation for Colorectal Cancer Patients: Study Protocol of a Nationwide Multicentre Study With Uniform Prehabilitation Protocols

**DOI:** 10.1002/wjs.12565

**Published:** 2025-04-02

**Authors:** C. R. Sabajo, J. P. Dieleman, J. W. T. Dekker, B. van den Heuvel, J. M. Klaase, G. D. Slooter

**Affiliations:** ^1^ Department of Surgery Máxima Medical Center Veldhoven the Netherlands; ^2^ Department of Surgery University Medical Center Groningen Groningen the Netherlands; ^3^ Science Office Máxima Medical Center Veldhoven the Netherlands; ^4^ Department of Surgery Delft the Netherlands; ^5^ Department of Surgery Radboud University Medical Center Nijmegen the Netherlands

**Keywords:** colorectal cancer, colorectal surgery, prehabilitation, uniform guidelines

## Abstract

**Background:**

The aim of prehabilitation is to optimize patient specific modifiable risk factors before major surgery, in order to enhance the individual resilience. Due to the lack of universal guidelines, prehabilitation has been conducted in various ways, making it difficult to estimate its effect. In the Netherlands, proposed uniform prehabilitation protocols were developed. The aim of this study is to analyse clinical outcomes of prehabilitation when implemented as standard of care according to the proposed uniform protocols.

**Methods:**

Uniform prehabilitation protocols were created based on the multimodal program of the PREHAB randomized controlled trial. All hospitals in the Netherlands that implemented prehabilitation according to the proposed protocols, for patients undergoing elective colorectal surgery, will be asked to participate. This study will recruit 535 patients who underwent prehabilitation and 535 one‐to‐one nearest neighbour propensity score matched patients who did not undergo prehabilitation (historical cohort). Clinical outcomes will be compared between the prehabilitation group and the historical cohort group using regression analyses. The primary outcome of interest is 90‐day presence of postoperative complications. In addition, length of hospital stay and readmissions will be analysed.

**Discussion:**

Prehabilitation has been proven to reduce the risk of complications and hospital length of stay. Prehabilitation has however been defined in various ways, since there is no standardized program. This multicentre cohort study will estimate the clinical effect of prehabilitation implemented as standard of care according to proposed uniform protocols. Furthermore, the presented protocols can be used by other hospitals to set up a prehabilitation program.

## Graphical abstract

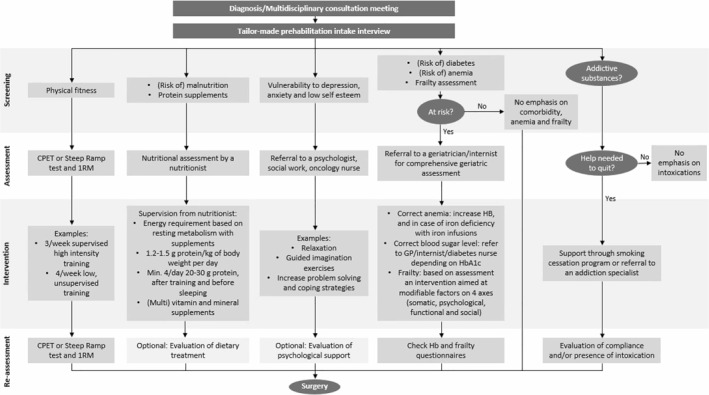

**Abbreviations**
1RM: One repetition maximum
6CIT: Six‐Item Cognitive Impairment test
CCI: Comprehensive Complication Index
CGA: Comprehensive geriatric assessment
CPET: Cardiopulmonary exercise testing
CRC: Colorectal Cancer
DCRA: Dutch ColoRectal Audit
HADS: Hospital Anxiety and Depression Scale
HIIT: High‐intensity interval training
HRmax: Maximum heart rate
IQR: Interquartile range
iSWT: Incremental shuttle walk test
MET: Metabolic equivalents
MMC: Maxima Medical Centre
NZa: Dutch health Authorities
PG‐SGA: Patient Generated Subjective Global Assessment
RCT: Randomised controlled trial
SRT: Steep Ramp Test
VO2max: Maximal oxygen consumption
VSAQ: Veterans‐specific activity questionnaire
Wpeak: Peak power output

## 1 Background

Prehabilitation is designed to enhance a patient's physical condition prior to a major surgery, resulting in faster recovery and fewer complications. The fitter the patient is before surgery, the greater the chance of a better outcome [1, 2]. The prehabilitation program is tailored to the individual and includes physical training, customized nutrition, mental support, comorbidities (e.g. diabetes) and anaemia optimization, and smoking and alcohol cessation.

While it has been demonstrated that a patient's functional capacity can be improved through prehabilitation [3, 4] it is not yet clear to what extent prehabilitation actually improves surgical outcomes. Due to the lack of uniform guidelines, the prehabilitation program has been implemented in different forms. In some studies, aerobic exercise in combination with resistance training was offered as prehabilitation [5, 6, 7] while in another study only yoga was offered [8]. Several systematic reviews also found a lot of heterogeneity within prehabilitation studies [9, 10, 11]. This heterogeneity makes it difficult to compare results between studies and depicts the need to develop uniform guidelines.

With the international randomized controlled PREHAB study [12] the development of proposed uniform guidelines had begun. The protocol has for example been used as a blueprint for other studies. In the Netherlands, the Dutch Society of Surgery developed uniform prehabilitation protocols based on the international PREHAB RCT [12]. These proposed uniform protocols were assessed and adjusted by relevant scientific associations of sport medicine, anaesthesiology, surgery, internal medicine, geriatrics, medical psychology, physical therapy and nutritionists. Furthermore, anaemia screening and management was added. The proposed protocols were created to ensure the quality of prehabilitation programs in the Netherlands, to eventually evaluate outcomes of prehabilitation in a bigger population, and to evaluate the feasibility of implementing such a program. The proposed uniform protocols describe the core points of the prehabilitation program for patients with colorectal cancer (CRC). They can be used by other hospitals, inside and outside the Netherlands, to set up prehabilitation.

This paper describes the proposed uniform protocols created by the Dutch Society of Surgery and study protocol to evaluate its clinical effects. The study includes a nationwide multicentre study, which aims to evaluate clinical outcomes of prehabilitation when implemented as standard care, using the presented proposed uniform protocols. Also, length of stay and readmissions will be analysed.

## 2 Methods

This nationwide multicentre observational cohort study will be conducted in several hospitals in the Netherlands that implemented the proposed uniform protocols and followed prehabilitation training facilitated by Maxima Medical Centre (MMC) and the foundation Fit4Surgery. Approval of the Medical Research Ethics Committee was requested but formal approval was deemed unnecessary, according to Dutch law (METC UMCG, 2022/079). The study procedure was approved by the Institutional Review Board of each participating hospital.

### 2.1 Study Population

Adult patients undergoing elective colorectal resection for cancer and participating in the prehabilitation program are eligible for inclusion into the intervention cohort. Patients from the same hospital but from 2 years before implementation of the proposed uniform protocols, will be eligible for inclusion into the reference cohort. The intervention and reference cohort will be matched one‐to‐one based on propensity scores.

### 2.2 Informed Consent and Data

All patients who underwent a prehabilitation program in one of the participating centres will be asked for informed consent. Based on local institutional review boards, patients will either be included upon providing written informed consent or upon applying the general opt‐out procedure. From every participating hospital, a historical cohort will be included to match with the prehabilitation group. The reference cohort will comprise patients from 2 years before when prehabilitation was not yet implemented in the respective hospital.

For both groups, data on patient characteristics, tumour and clinical outcome will be obtained from a nationwide quality registration database, the Dutch ColoRectal Audit (DCRA). The collected prehabilitation data will be combined with the DCRA data, based on date of birth, sex and operation date from the patient.

### 2.3 The Prehabilitation Program

The prehabilitation program as described by the proposed uniform protocols includes the following five modalities.

– Physical fitness
– Nutritional status
– Mental resilience
– Comorbidity, anaemia, and frailty
– Intoxications

The prehabilitation program should last at least 3–4 weeks to see an effect from the program. A longer prehabilitation period does not seem to have a negative effect on oncological treatment [13, 14]. If a patient can undergo surgery before the prehabilitation program is completed, it is preferable to complete the prehabilitation program first. Also after the surgery a healthy lifestyle is encouraged. A visit to a physiotherapist postoperatively (> 6 weeks) is preferred, where physical fitness measurements are repeated. Ideally, prehabilitation should be linked to rehabilitation, and these should be well integrated.

The flow chart in Appendix 1 shows the framework of screening, assessment, intervention, and re‐assessment for each modality in the prehabilitation program. Shared decision‐making is an integral part of all steps in the prehabilitation process.

### 2.4 Interventions (According to the Proposed Uniform Protocols)

A more detailed overview of the interventions can be found in the appendices.

1. Physical fitness (Appendix 2)
  - Endurance training: Three times a week supervised high‐intensity interval training (HIIT) under the supervision of an (oncological) physiotherapist. Low‐intensity training at home on the other days. The training program is individualized and is based on the initial physical fitness assessment.
2. Nutritional status (Appendix 3)
  - Nutritional consultation with a dietitian: Aim for 1.2–1.5 g of protein per kg of body weight per day and energy intake according to calculated energy needs. Protein supplementation or medical nutrition can be used to achieve recommendations. Supplementation of vitamins and minerals is recommended.
3. Mental resilience (Appendix 4)
  - Providing information to the patient, taking into account the patient's health literacy, for example by a case manager.
  - Psychological support: Consultation with case manager or specialized nurse. Referral to a medical psychologist in case of anxiety or depression.
4. Comorbidity, anaemia, and frailty (Appendix 5 and 6)
  - Blood sugar level: Measure HbA1c. Refer patients without diabetes with a threshold value > 42 mmol/mol (6%) to GP/internist. Refer patients with non‐insulin‐dependent diabetes with a threshold value > 53 mmol/mol to GP/diabetes nurse.
  - Anaemia: Optimize haemoglobin levels if the value is < 8.0 mmol/L in men and < 7.5 mmol/L in women. Optimize iron deficiency preferably with intravenous treatment (Appendix 6).
  - Frailty: Referral to a geriatrician in case of frailty based on, for example, the Groningen Frailty Indicator, G8 questionnaire, 6CIT questionnaire, and/or MiniCog or if a delirium has occurred previously.
5. Intoxications (Appendix 7): Support for smoking cessation and cessation of other addictive substances.

### 2.5 Study Outcomes

The primary outcome will be 90‐day postoperative complications. This will be graded according to Dindo et al. [15] and defined as presence or absence of any complication. Complications will also be scored with the Comprehensive Complication Index (CCI) [16]. A CCI > 20 will be labelled as a severe complication. Secondary outcomes will be length of hospital stay and readmissions.

Data on study outcomes is available in the DCRA database, which is a national mandatory audit. Prehabilitation data, for example start date of prehabilitation, number of training days, and outcomes of physical tests, will be collected separately and combined with the data from the DCRA.

### 2.6 Statistical Analysis

A sample size calculation was made based on multiple logistic regression analysis for presence of post‐operative complications within the 90 postoperative days. Age, ASA score, Charlson Comorbidity score, BMI, type of surgery, and surgical approach will be considered as potential confounders. For the sample size calculation the effect size was based on the PREHAB study [12], with a two‐sided *p*‐value for significance of 0.05 and a desired power of 80%. To show a reduction in overall complication rate from 42.2% to 31.7% (odds ratio (OR) = 0.62) and being able to adjust for the above confounding factors, 535 patients are needed in each group, resulting in a total required study population of 1070 patients.

One‐on‐one propensity score matching will be performed to control for potential confounders. Propensity score matching will be achieved per hospital. Propensity scores will be calculated using multiple logistic regression analysis including, age, ASA score, Charlson Comorbidity score, BMI, type of surgery, and surgical approach as the independent variables and prehabilitation (yes/no) as the dependent variable. The resulting probabilities will be used to match each patient in the prehabilitation cohort with its nearest neighbour in the reference cohort. In case of multiple matches, a reference patient will be randomly selected. The effect of larger distances will be explored in sensitivity analyses.

Baseline characteristics and clinical outcomes will be presented for the prehabilitation and reference cohort. Continuous variables will be presented as mean (SD) and will be tested using the unpaired *t*‐test if there was a normal distribution based on the skewness and/or kurtosis value and presented as mean (SD). If a not normal distribution is nor present, the Mann–Whitney *U* test will be used and presented as median with interquartile range (IQR). Categorical variables will be presented as number per category with percentages and will be analysed using the Chi‐square or Fishers' exact test as appropriate. To calculate ORs and regression coefficients with 95%‐confidence intervals (95%CI), logistic and linear regression analyses will be used. Data will be analysed on an intention‐to‐treat basis. Per protocol analyses excluding patients who underwent insufficient prehabilitation will be done to explore the effect of prehabilitation compliance. The amount of missing data will be compared between the two study cohorts; baseline characteristics will be compared between patients with and without missing's to explore the possibility and direction of bias through missing's.

## 3 Discussion

This multicentre cohort study will include real‐world data from hospitals in the Netherlands, where prehabilitation has been implemented according to the proposed uniform protocols for CRC patients and if hospitals followed the prehabilitation training provided by MMC and the foundation Fit4Surgery.

Over the years, an increasing number of studies have been performed to evaluate the effect of prehabilitation. Several small trials showed less complications and/or reduced hospital length of stay due to prehabilitation [4, 5, 6, 17]. In addition, Barberan et al. [18] performed a RCT using a structured program, and showed that prehabilitation was a protective factor for postoperative complications in 76 CRC patients that underwent elective surgery. Klerk et al. [17] performed an RCT also with a standard program, in patients undergoing elective resection for CRC. They showed that prehabilitation significantly reduced overall postoperative medical complications, unplanned readmissions, and the median length of hospital stay. Berkel et al. [19] showed an almost 50% decrease in the incidence of postoperative complications due to prehabilitation for CRC patients in a selected group of patients with high risk of complications (anaerobic threshold < 11 mL/kg/min). Heil et al. also found in their emulated target trial a relevant reduction of complications and length of hospital stay in patients with a higher postoperative complication risk [20]. The international PREHAB trial used an uniform multimodal program in both high risk and non‐high risk patients and showed significantly lower severe complications, and medical complications in the prehabilitation group [12].

In contrast to these previous studies, which performed prehabilitation in a study setting, outcomes of a prehabilitation program implemented as standard of care for CRC patients are still lacking. A previous singe centre study by Sabajo et al. [21] showed that prehabilitation in CRC surgery improves outcome and reduces hospital costs. Since the effectivity of prehabilitation has to be evaluated on a larger scale, this study will evaluate the outcomes of proposed uniform protocols for a multimodal prehabilitation program for CRC resections when implemented as standard of care throughout multiple hospitals in the Netherlands. Therefore, the provided uniform guidelines were created to enhance standardization and uniformity of the program. Prehabilitation is a multidisciplinary intervention, which can make it difficult to implement the program within a hospital. The proposed protocols might help hospitals to implement prehabilitation, since all involved interventions are described in detail. Hospitals in the Netherlands will receive training in how to implement prehabilitation with an optional additional training for physiotherapists. Hospitals can get reimbursement through the “Innovation Rule” initiated by the Dutch health Authorities (NZa) depending on the insurance company.

In comparison to the protocol used in the PREHAB trial [12] optimization of anaemia has been added. The LekCheck study found that low preoperative haemoglobin was the most important factor involved in the development of an anastomotic leakage [22]. Also, Berkel et al. found that a lower preoperative haemoglobin level was a risk for the development of complications [19]. Furthermore, the FIT trial found that with the treatment of intravenous iron deficiency a significant improvement was seen in the preoperative haemoglobin level [23]. Subsequently, the optimization of anaemia with intravenous iron was added to the proposed uniform protocols.

Due to its nature, this study is a non‐randomized, non‐blinded study. However, since prehabilitation is, and will be, implemented as standard of care, data will be real‐world data. Given the promising results of prehabilitation, a RCT is no longer suitable. It would not be ethical to withhold high‐intensity training, mental support and nutritional, frailty and anaemia screening, assessment and interventions from patients.

This paper presents the first standardized proposed uniform protocols for prehabilitation for CRC patients who will undergo elective surgery. The program consists of supervised high‐intensity training, nutritional assessment with protein and vitamin supplements, mental health support, assessment of comorbidities and frailty, anaemia screening and optimization and cessation of smoking. Further, this paper presents the protocol of a nationwide multicentre study, which aims to evaluate clinical outcomes of prehabilitation when implemented as standard care, using the proposed uniform protocols.

## Author Contributions

**C. R. Sabajo:** conceptualization, methodology, writing – original draft. **J. P. Dieleman:** methodology, writing – review and editing. **J. W. T. Dekker:** conceptualization, writing – review and editing. **B. van den Heuvel:** conceptualization, writing – review and editing. **J. M. Klaase:** conceptualization, supervision, writing – review and editing. **G. D. Slooter:** conceptualization, supervision, writing – review and editing.

## Ethics Statement

The Medical Ethics Committee of the Medical University of Groningen approved this study.

## Conflicts of Interest

The authors declare no conflicts of interest.

## Data Availability

The data regarding clinical outcomes supporting the findings of this study are available on the corresponding author on reasonable request.

## Appendix 2 Physical Fitness

### Definition and Goals

Major colorectal surgery can lead to substantial morbidity and mortality, particularly in elderly and patients with comorbidities. Low cardiorespiratory fitness has been found to be a major risk factor for complications after colorectal surgery [24]. Cardiorespiratory fitness is the maximum capacity of the body to take up oxygen and to transport it to the tissue [25]. It's expressed as VO2max (in mL/kg/min body weight) or metabolic equivalents (METs; where 1 MET is equal to the resting metabolism with an oxygen uptake (VO2) of about 3.5 mL/kg/min).

The goal is to make a preoperative risk assessment and to optimize the patient's cardiorespiratory fitness and muscle strength to reduce the risk of perioperative complications and to accelerate postoperative recovery of physical functioning.

#### Preoperative Risk Assessment

Cardiopulmonary exercise testing (CPET) is the gold standard for measuring cardiorespiratory fitness [24, 25]. It is increasingly used preoperatively with a subsequent risk assessment. Several studies show that patients have an increased risk of complications at VO2max < 16–18 mL/kg/min and/or a VO2 < 10–11 mL/kg/min at the ventilatory threshold (first threshold) [19, 24, 26, 27]. In a large cohort with CPET variables and BMI, patients with a VO2 at the ventilatory threshold ≤ 11.1 mL/kg/min had an odds ratio (OR) of 7.56 for complications during hospitalization after colorectal surgery, where this OR was 2.15 at a VO2max ≤ 18.2 mL/kg/min [1]. However, a CPET is relatively time‐consuming and requires medical supervision. Therefore, it does not seem cost‐effective to administer a CPET preoperatively to every patient. In addition, a CPET is not available in every centre. With questionnaires, such as the “FitMáx" [28] or “veterans‐specific activity questionnaire” (VSAQ), an estimate of the cardiorespiratory fitness can be made, after which only patients who score low undergo a CPET. In various studies, a cut‐off value of ≤ 7 METs on the VSAQ was used for this purpose [19, 26]. In addition to a risk assessment, the CPET can also map (the severity of) cardiovascular and pulmonary comorbidities, which can then be adjusted for pre‐ or perioperative management [29]. If a CPET is not feasible, it is advisable to use an alternative maximal test for risk assessment (e.g., the steep ramp test (SRT) or incremental shuttle walk test (iSWT)). Although a better SRT and iSWT performance is associated with better postoperative outcomes [30], an accurate cut‐off point is not yet available for these tests.

#### Interventions

Every patient is informed about the importance of physical activity and physical fitness. Additionally, it is recommended to optimize the physical fitness of especially those patients with cardiopulmonary fitness, according to the CPET threshold values described above, preoperatively under the supervision of a (oncological or geriatric) physiotherapist specifically trained for prehabilitation. It is important to take into account the patient's wishes and capabilities. High‐intensity interval training (HIIT) appears to be the most effective way to improve cardiorespiratory fitness (and muscle strength) in a short period (4–6 weeks). In a RCT, preoperative training for high‐risk patients prior to colorectal surgery resulted in improved physical fitness after 3 weeks, which subsequently led to a significant reduction in the number of patients with postoperative complications [19].

There are many variations possible in the design of a preoperative physical training program. Intensity can be varied in different ways.

#### High‐Intensity Interval Training

High‐intensity training program according to the adapted Meyer protocol [31] as described in van Wijk et al. [32].

- Particularly suitable for frail patients
- Baseline measurement: adapted SRT protocol (2 min unloaded cycling as warm‐up, followed by a load increase of 10 W (W) every 10 s until exhaustion (pedal frequency drops < 60 rpm) despite verbal encouragement, followed by a cool‐down. The highest achieved load (Wpeak) is the primary outcome measure of the SRT)
- 3–5 times a week 15–25 min SRT‐based HIIT on a cycle ergometer
  ◦ 3 min warm‐up at 20 W
  ◦ 30 s work interval at 60% of the SRT Wpeak
  ◦ 60 s rest interval at 20 W
  ◦ 8–14 intervals
  ◦ Cooling down for at least 1 min after the last rest interval
  ◦ Based on the clinical judgment of the physiotherapist and/or the degree of experienced effort of the patient (ideally Borg scale 15–18 on the range 6–20), the load during the work intervals can be adjusted
- Measuring/monitoring progression: the SRT is repeated weekly to maintain the intensity at the right level

High‐intensity training program according to the Wisløff protocol [33]as described in van Rooijen et al. [34].

- Particularly suitable for the non‐frail patient
- Baseline measurement: maximum effort test, preferably CPET, to determine maximum heart rate (HRmax)
- 3 times a week a 38 min HIIT training on a cycle ergometer, treadmill, or rowing ergometer
  ◦ 6 min warm‐up at 60%–70% of HRmax
  ◦ 4 min work interval where the last 2 min should reach 90%–95% of HRmax (set/adjust the load of the work interval accordingly)
  ◦ 3 min rest interval where HR should drop to 50%–70% of HRmax
  ◦ 4 intervals
  ◦ 4 min cooling down at 50%–70% of HRmax
  ◦ Based on the clinical judgment of the physiotherapist and/or the degree of experienced effort of the patient (Borg 15–18), the load during the work intervals can be made slightly less or more difficult.

#### Muscle Exercises

It is recommended to pay attention to (functional) muscle strength training in addition to improving cardiorespiratory fitness, in order to ensure the patient's mobility in daily life and facilitate a (sustainable) active lifestyle. Especially for frail patients, it is advisable to do this through functional exercises that can be incorporated into the patient's daily life and can be performed at home, such as those described in Van Wijk et al. [32].

- Chair squats/getting up from a chair
- Stair climbing/steps
- Lifting (groceries/boxes)

Especially for the non‐frail patients, specific muscle groups can be trained by a physiotherapist, as described in Berkel et al. [19] and Van Rooijen et al. [34].

- 3 times a week exercises for different muscle groups
  ◦ For example, leg press, chest press, abdominal crunch, lateral pull down, low row, and/or step up
  ◦ 2 sets of 10 repetitions or 3 sets of 8 repetitions
  ◦ Intensity based on the calculated 1RM at baseline (e.g., using the Oddvar‐Holten diagram)
    - Week 1: 65% of calculated 1RM
    - Week 2: 70% of calculated 1RM
    - Week 3: 75% of calculated 1RM
    - Week 4: 80% of calculated 1RM
  ◦ The last set of 10 repetitions should be achievable for the patient.
    - If not, the intensity should be decreased by 5%–10% in the subsequent training
    - If the last set of 10 repetitions is too easy, the intensity should be increased by 5%–10% in the subsequent training

##### Breathing exercises

It is recommended to meet patients undergoing abdominal surgery preoperatively to instruct them on breathing and breathing exercises. This reduces the risk of postoperative pulmonary complications. Preoperative breathing exercises supervised by a physiotherapist have a greater effect on preventing pulmonary complications than postoperative physiotherapy alone [35].

This preoperative physiotherapy session includes.

- Identifying risk factors (smoking, lifestyle, pulmonary complications)
- Teaching breathing techniques, consisting of:
  ◦ 10 deep maximum inspirations, followed by holding the breath for 2–3 s
  ◦ Teaching coughing techniques
  ◦ Assessing cough strength using peak cough flow

## Appendix 3 Nutritional Status

### Definition and Objectives

The preoperative dietary treatment aims to improve a patient's poor nutritional status or to maintain a good nutritional status and to increase the effect of strength training [36]. This way, patients enter surgery as fit as possible, improving their chances of recovery and reducing the risk of complications.

#### Triage

Triage determines which nutritional care is necessary within the prehabilitation process for every patient scheduled for surgery. Since patients with low functional reserve and low muscle mass (such as the elderly, patients with sarcopenia, and patients with an oncological condition [37, 38]) likely benefit the most from prehabilitation, screening for (the risk of) malnutrition [38, 39] and protein intake is performed. For patients with an oncological condition, the PG‐SGA SF [40, 41, 42, 43] is recommended. Patients at risk for malnutrition or with a PG‐SGA core of four or more should always be referred to a dietician for tailored advice.

The Dutch Dietary Guidelines are followed for every patient. In addition, sufficient protein intake is emphasized to increase the effect of strength training. If desired, these general recommendations for patients (without risk of malnutrition) can be given by other healthcare providers as well (in consultation with the dietician).

#### Nutritional Assessment by Dietician

After screening identifies a risk of malnutrition, the dietician performs a nutritional assessment. Nutritional assessment systematically evaluates and defines the nutritional status and nutritional needs to develop an adequate treatment plan [44]. The European Society of Clinical Nutrition and Metabolism (ESPEN) has defined five nutrition‐related disorders and conditions [45]. These include malnutrition, sarcopenia and frailty, overweight and obesity, micronutrient deficiencies, and refeeding syndrome. Malnutrition and sarcopenia can be further categorized by cause and severity [46, 47, 48, 49]. [44]The assessment of nutritional status and nutritional needs is based on history‐taking and measurements. These can be categorized into three domains: “food intake, utilization and losses,” “body composition and nutrient reserves,” and “functional parameters” [44].

#### Interventions

##### Dietary Treatment by a Dietician

The optimal dietary treatment should be determined on an individual level. The following recommendations are made for preoperative optimization of the patient's condition.

- The patient's total energy requirement consists of a combination of resting metabolism and a supplement factor for activity and illness. To determine resting metabolism as accurate as possible, it is preferred to measure it using indirect calorimetry. If this is not feasible, resting metabolism can be estimated using the WHO or Harris and Benedict (1919) formula. An estimate should also be made for any energy losses that may occur [38, 50, 51, 52, 53].
- Exercise in combination with sufficient protein intake is essential to strengthen muscles. For prehabilitation, 1.5 g of protein per kg fat free mass is considered as minimum, with 1.9 g of protein per kg fat free mass being recommended (either 1.2–1.5 g of protein per kg body weight) [1, 54, 55, 56, 57, 58].
- Timing of meals affects muscle protein synthesis related to strength training. Therefore, it is recommended to use 20–30 g of protein (of high quality) within 30–60 min after strength training. Additionally, for a maximum protein synthesis, it is recommended to use at least four servings of 20–30 g of protein per day, starting with breakfast and the last serving 30 min before bedtime [36, 58, 59, 60, 61, 62].
- If possible, the Dutch Dietary Guidelines are followed as much as possible. However, in cases of complex nutritional problems and poor nutritional status, the dietary advice may not always correspond to these guidelines. Supplementation of vitamins and minerals is only necessary in case of deficiencies and when intake is not or insufficiently possible. In that case, supplementation up to a maximum of 100% of the recommended daily needs is recommended. Vitamin D intake requires extra attention according to the advice of the Health Council (10–20 μg/day) [63, 64, 65].

If necessary, protein supplementation or medical nutrition can be used to meet the above recommendations.

#### Evaluation of Dietary Treatment

After the start of the dietary treatment in a prehabilitation program, it is recommended to evaluate the nutritional status [39]. The content, duration, and frequency of dietary treatment and evaluation moments vary per target group and per patient. Various factors play a role in the choice of measurements that are carried out for follow‐up purposes, such as the severity of malnutrition, the patient's ability to cope, costs, and the extent to which an instrument can measure a real change (responsiveness) [38]. Measures in the domain of food intake and losses are particularly suitable for shorter‐term follow‐up measurements. However, more time is needed to measure relevant changes in body composition and functioning [66].

## Appendix 4 Mental Resilience

### Definition and Goals

Mental resilience refers to the cognitive, emotional, and social skills that enable a person to lead a meaningful and productive life and to successfully fulfil different social roles and functions during different stages of their life [67]. The goal of increasing mental resilience for patients in prehabilitation is to increase coping skills to deal with the recently diagnosed cancer. Reducing possible anxiety and depressive symptoms leads to a greater perceived quality of life [68].

#### Interventions

Interventions described in the literature mainly concern: (1) Relaxation (breathing and progressive muscle tension exercises), (2) Guided imagery exercises (imagining periods before and after surgery and picturing a safe place) and (3) Increasing problem‐solving and coping strategies. There are varying effects of perioperative psychological interventions described in the literature. The effects that are found are mainly visible on patient‐reported outcome measures such as quality of life, anxiety and depressive symptoms, and self‐efficacy. No effects were found on surgical outcomes such as length of stay and complications [69].

It seems particularly important to identify patients who are vulnerable for depression, anxiety, and low self‐efficacy [68]. This can be screened, for example, by the HADS. Ideally, these outcomes should be evaluated and discussed by an oncology nurse or nurse specialist. If necessary, a referral can then be made to a psychologist or medical social worker.

In two prehabilitation pilots conducted at the Máxima Medical Centre (Veldhoven, the Netherlands) for both CRC and lung cancer patients, it was not necessary to have all patients seen by a psychologist. A better indication is therefore the score on the screening instruments and a possible referral after the conversation with the oncology nurse (specialist). Then, a personalized offer of interventions can be considered [70]. It is possible that in the future, more clarity will be gained about which psychological interventions are effective for prehabilitation.

## Appendix 5 Frailty and Comorbidity

### Vulnerability

#### Definition and Objectives

Frailty is the reduction of reserve capacity in different physiological systems, in which these reserves have dropped to a critical minimum [71]. A small disturbance of this unstable physiological balance can lead to one or more geriatric syndromes, such as functional decline, falls, confusion, and incontinence. This involves a strong interdependence of the biological, psychological, and social domains. A change in one domain may have consequences for another domain. A geriatric syndrome is the result of several conditions occurring simultaneously and implies a multi‐causal complaint or symptom [72]. Frailty arises from a build‐up of effects of aging, lifestyle factors, and diseases that manifest during life. Vulnerability is often subclinical and is not infrequently exacerbated by an acute condition or surgery and can subsequently lead to functional loss and complications. There is no gold standard for frailty screening. Instruments range from multidomain (GFI, TFI, G8, etc.) to monodomain.

The aim is to make an estimate of the extent to which the patient can withstand the intended treatment, or is at risk of complications, functional loss, and mortality. In addition, the aim is to identify potentially modifiable characteristics of frailty and try to optimize them as much as possible to minimize the risks mentioned above.

#### Interventions

- In case of positive identification by screening (e.g., GFI > 4; G8): referral to a clinical geriatrician/internal medicine physician specialised in geriatrics for a comprehensive geriatric assessment (CGA) [73, 74].
- Based on the CGA, interventions focused on modifiable factors on the four axes (somatic, psychological, functional, and social):
  ◦ Sarcopenia, reduced mobility/high fall risk
  ◦ Malnutrition
  ◦ Unidentified comorbidities
  ◦ Polypharmacy and high‐risk medication
  ◦ Delirium risk
  ◦ Affective problems/psychiatric comorbidities
  ◦ Decreased self‐reliance (attention to small social network/living alone)

### Comorbidity

#### Definitions and Objectives

Comorbidity is defined as the presence of (chronic) comorbid conditions such as chronic heart failure, COPD, diabetes, dementia, etc., also indicated with a comorbidity index [75].

The aim is to make an estimate of the possible risks that the patient faces with a planned treatment and to weigh them against the risks of CRC and CRC treatment. It is also an opportunity to optimize the comorbid disease as much as possible in order to minimize the risk of complications or other negative outcomes.

#### Interventions

- Estimate mortality risk and current risk factors, based on for example the CCI, ASA classification and/or vulnerability, and weigh them against the risk of colorectal malignancy and the treatment deemed necessary
- Treat the identified comorbid disease as much as possible
  ◦ Diabetes Mellitus: Measure HbA1c if glucose levels are elevated. Refer to a general practitioner/internist for patients:
    – Without diabetes with a threshold value > 42 mmol/mol (6%)
    – With non‐insulin‐dependent diabetes with a threshold value > 53 mmol/mol (7%)
    – With insulin‐dependent diabetes < 10 years with a threshold value > 58 mmol/mol (7.5%)
    – With insulin‐dependent diabetes ≥ 10 years with a threshold value > 65 mmol/mol (8.1%)

## Appendix 6 Protocol for Correction of Iron Deficiency

### Definitions and Objectives

About one third of patients with CRC have anaemia [76]. In most cases, this concerns malignancy‐related iron deficiency anaemia, which is multifactorial in nature (amongst others due to gastrointestinal bleeding, inflammation, and nutritional deficiencies) [77]. In some cases, anaemia may have a different cause, such as chronic kidney disease, haemolysis, or bone marrow infiltration.

Preoperative anaemia is associated with fatigue [78] perioperative blood transfusions [79], postoperative morbidity and mortality [22, 80, 81] and reduced survival [82, 83]. Preoperative anaemia screening and treatment are therefore recommended [84].

Anaemia according to the World Health Organization [85].

- Haemoglobin < 8.0 mmol/L for men
- Haemoglobin < 7.5 mmol/L for women

The objective is to preoperatively optimize the haemoglobin concentration in patients with colon cancer. In other words, the aim is to increase haemoglobin levels if they are < 8.0 mmol/L in men and < 7.5 mmol/L in women.

Diagnostic procedure (see Flowchart below).

The diagnostic procedure should be requested as early as possible, for example immediately after colonoscopy by the gastroenterologist or case manager.

The diagnostic procedure consists of the following lab tests.

- Hb (mmol/L)
- MCV (fL)
- Iron (μmol/L)
- Ferritin (μg/L)
- Transferrin (g/L)
- Transferrin saturation (TSAT) (%)
- CRP (mg/L)

Iron deficiency can be subdivided into two categories; absolute and functional iron deficiency [77]. In both cases, preoperative administration of intravenous iron is advised (see flowchart below) [84, 86]. If there is no iron deficiency, a consultation by an internist can be considered for further diagnostic evaluation. If the patient has already been referred to a geriatrician/specialist in elderly care, no separate referral to the internist is necessary.

### Intervention for Iron Deficiency

Treatment with 1000 mg Ferinject (ferric(III)carboxymaltose) takes place in the outpatient clinic according to local hospital procedures. The use of Ferinject (iron(III)carboxymaltose) is recommended, because of the lower risk of allergic reactions compared to other intravenously administered iron‐containing drugs.

### Monitoring the Effectiveness of the Intervention

To monitor the treatment effect, haemoglobin is again determined preoperatively (+/− 3 weeks after administration of Ferinject).

Note: The aim of this protocol is to optimize the haemoglobin level. This does not mean striving for a haemoglobin level of ≥ 8.0 mmol/L for men or ≥ 7.5 mmol/L for women with transfusions and/or other blood products.

## Appendix 7 Intoxications

### Definitions and Goals

Intoxications refers to the use of alcohol, nicotine, or other addictive substances, and polypharmacy. Polypharmacy is particularly relevant in vulnerable elderly patients and is screened and addressed through frailty screening. Attention is paid to high‐risk medication (e.g. lithium/digoxin use with narrow therapeutic range). With regard to the use of alcohol, nicotine, or other addictive substances, the advice during prehabilitation is to quit. This is discussed with the patient during a screening or intake. It will then also be determined if additional help is needed. Additional help will be organized differently in each hospital. There can be a clinic in the hospital, a referral to an external help organization and sometimes the general practitioner plays a role in this. In any case, the time perspective of prehabilitation should be taken into account and in general it is preferred to quit as quickly as possible.

### Interventions

#### Nicotine

Nicotine usage is related to the occurrence of postoperative complications [87, 88]. Although quitting smoking is always worth it, the earlier someone stops smoking preoperatively, the better. Nicotine replacement therapy is often used in combination with counselling. Depending on local arrangements, help and guidance will be organized. If this guidance is outsourced, ensure that there is an urgent referral given the time frame of the preoperative period.

#### Alcohol

The use of alcohol is, amongst others, related to an increased risk of postoperative delirium [89]. During screening or intake, the patient is advised to quit using alcohol. If there is addictive behaviour, a patient can be referred to an addiction medicine physician.

#### Other Addictive Substances

During screening or intake, the patient is also advised to quit using other addictive substances. If there is addictive behaviour, a patient can be referred to an addiction medicine physician.
